# Nonlinear van’t Hoff Behavior in the Interaction of Two Water-Soluble Porphyrins with Bovine Serum Albumin (BSA)

**DOI:** 10.1021/acsomega.4c07367

**Published:** 2024-11-20

**Authors:** Fabio C. Bezerra, Ernanni D. Vieira, Pablo J. Gonçalves, Iouri E. Borissevitch

**Affiliations:** † Instituto de Física, Universidade Federal de Goiás, Goiânia, Goiás 74690-900, Brazil; ‡ Programa de Pós-Graduação em Química, Instituto de Química, Universidade Federal de Goiás, Goiânia, Goiás 74690-900, Brazil; § Centro de Excelência em Hidrogênio e Tecnologias Energéticas Sustentáveis (CEHTES), Goiânia, Goiás 74690-900, Brazil; ∥ Departamento de Física, Faculdade de Filosofia, Ciências e Letras de Ribeirão Preto, Universidade de São Paulo, Ribeirão Preto, São Paulo 14040-900, Brazil

## Abstract

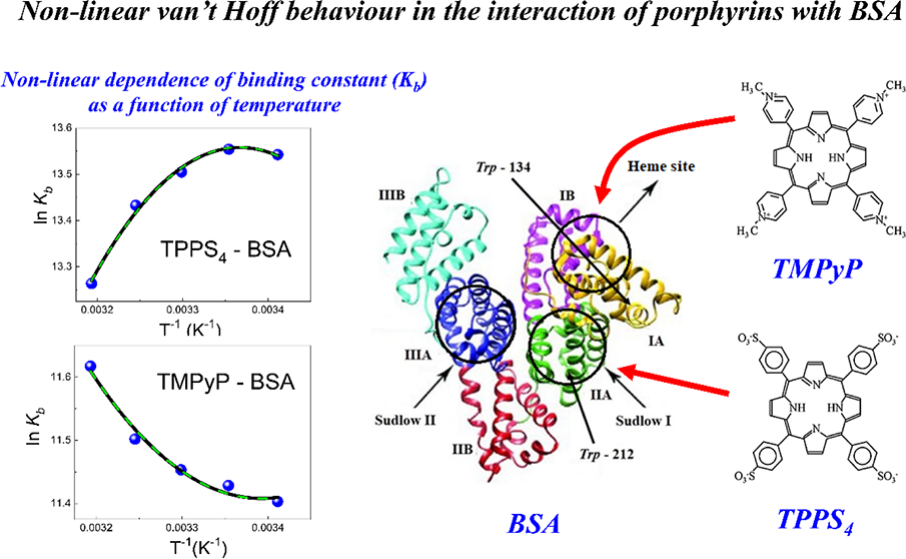

Thermodynamic analysis of the binding process of water-soluble negatively charged *meso*-tetrakis(*p*-sulfonatophenyl) (TPPS_4_) and positively charged *meso*-tetrakis(4-methylpyridyl) (TMPyP) porphyrins with bovine serum albumin (BSA) at different temperatures was carried out based on the data of BSA quenching fluorescence by porphyrins. The comparison of binding constants (*K*_b_) shows that negatively charged TPPS_4_ possesses higher affinity to BSA than positively charged TMPyP. Thermodynamic characteristics of the binding process were obtained in accordance with the van’t Hoff theory by processing nonlinear dependences of ln *K*_b_ on inverse absolute temperature within the framework of two models: taking into account the dependence or independence of the change in the standard heat capacity (Δ*C*^0^) on temperature. A comparison of thermodynamic characteristics with the data obtained from the Förster fluorescence quenching theory and with literature data leads to the conclusion that TPPS_4_ is bound to the Sudlow I site (subdomain IIA), while TMPyP is bound to the Heme site (between the subdomains IA and IB). The analysis of Δ*C*^0^ changes with temperature demonstrates that binding of TPPS_4_ promotes hydration of nonpolar groups in the protein, which increases with the increase of temperature, while binding of TMPyP decreases the hydration of polar groups of the protein, the effect increasing with rising temperature. The obtained information may be useful for elucidating the mechanisms of interaction of porphyrins with albumins and the effect of this interaction upon the effectiveness of porphyrins in photodynamic therapy and in fluorescence diagnostics of cancer.

## Introduction

Photodynamic therapy (PDT) is a technology for treating various diseases, including cancer, which has been rapidly developing in recent years. The principle of PDT is to introduce a photoactive compound (photosensitizer or PS) into the organism of a patient and activate it at the site of treatment with visible or near-infrared light. Excited states of PS, interacting with molecular oxygen, form reactive oxygen species (ROS), which kill cells of treated tissues.^1−3^ When PS is fluorescent, it can be used simultaneously with treatment as a fluorescent probe (FP) in fluorescent diagnostics (FD).^4−6^

Porphyrins and porphyrin derivatives are widely applied as PS for PDT and FP for FD due to their characteristics: intense optical absorption in the visible and near-infrared spectral regions, high quantum yields of ROS production, and relatively intense fluorescence.^4−6^

To be effective for both PDT and FD, it is necessary that PS be localized predominantly in the tissues to be treated. In other words, it must have a high affinity to these tissues. Some porphyrins have elevated affinity to malignant tissues; however, the difference of their affinity to malignant and normal tissues is not very large. Moreover, this difference depends on the type of tissue and the structure of the porphyrin.

The drug delivery technique is widely used to increase the PS specific affinity for diseased tissues. Among drug delivery systems, serum albumins are of particular interest as (1) they are natural intrinsic proteins present in serum at high concentrations (≈35–50 g/L); (2) they have a special function of binding various compounds (intrinsic and extrinsic) and transporting them through the organism with the bloodstream; (3) they demonstrate a high affinity to malignant tissues.^7,8^ Thus, the binding of porphyrin to the albumin can increase the affinity of the porphyrin to diseased tissues. On the other hand, binding of PS to albumin can affect the characteristics of its excited states and the probability of reactions between excited PS molecules and molecular oxygen, thereby affecting the production of ROS and, consequently, the effectiveness of treatment. This effect should depend on the localization of the PS molecule in the albumin structure.

There are a lot of studies on the kinetic behavior of various porphyrins when interacting with albumins and of the effects of this interaction on photophysical properties of porphyrins. It was shown that interaction depends on the porphyrin structure, including the nature of side groups and the porphyrin charge state.^8−14^ This interaction affects the lifetime and quantum yield of the porphyrin excited states,^9−13^ thus affecting the quantum yield of the ROS formation. However, there are currently relatively few studies on the thermodynamic characteristics of porphyrin interactions with albumins and the effect of the porphyrin structure on these characteristics. At the same time, the analysis of these characteristics can provide important information about the mechanisms of interaction between porphyrins and albumins.

The ability of albumins to emit an intense fluorescence enables their fluorescence quenching as an effective technique for determining the characteristics of their interaction with other compounds, porphyrins, in particular.^10,15,16^ The determination of thermodynamic parameters for interaction using fluorescence quenching is based on the van’t Hoff analysis of the logarithm of the Stern–Volmer quenching constant, ln(*K*_SV_), as a function of the inverse absolute temperature (1/*T*). However, in many cases, the plots of ln(*K*_SV_) versus 1/*T* have been considered linear or a classical van’t Hoff plot, even when the fit of the experimental data was not satisfactory.^17−20^ The deviation of the van’t Hoff plots from linearity is directly related to the dependence of thermodynamic parameters on temperature.^21−26^ In this case, changes in the standard enthalpy and entropy are temperature-dependent. Additionally, the temperature dependence of the standard heat capacity plays an important role in interpreting the types of interactions involved in the protein binding process.^27,28^

To the best of our knowledge, no studies have utilized fluorescence quenching assays to evaluate the magnitude and behavior of thermal capacitance in protein–ligand interactions. Therefore, in this study, we analyzed the plots of ln(*K*_SV_) as a function of 1/*T*, considering both the temperature dependence and independence of heat capacity changes.

Water-soluble negatively charged *meso*-tetrakis(*p*-sulfonatophenyl) (TPPS_4_) and positively charged *meso*-tetrakis(4-methylpyridyl) (TMPyP) porphyrins were used as model systems to study the thermodynamic characteristics of their interaction with bovine serum albumin (BSA). The choice of these porphyrins is based on the fact that they have been already tested as PS, demonstrating high potential for PDT and FD applications^29−33^ and their photophysical properties are well studied.^34−37^ The main interest in the comparative study of these porphyrins is due to their water-solubility and their different electrical charges, which can improve interaction with different binding sites in endogenous proteins in the bloodstream.

## Materials and Methods

**Figure 1:**
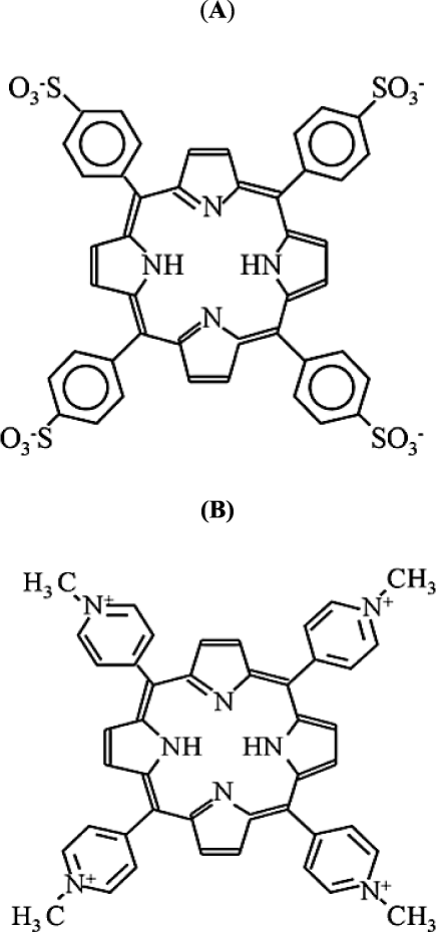
Structures of porphyrins TPPS_4_ (A) and TMPyP (B).

*meso*-Tetrakis(*p*-sulfonatophenyl) (TPPS_4_) and *meso*-tetrakis(4-methylpyridyl) (TMPyP) porphyrins (Figure 1) were purchased from Porphyrin Products Inc. Bovine serum albumin (BSA) was obtained from Sigma-Aldrich and used as purchased. The experimental solutions were prepared in phosphate buffered saline (PBS, pH 7.4). The compound concentrations were controlled spectrophotometrically using the molar absorption coefficients ,^33^ ,^33^ and .^38^

UV/vis absorption measurements were performed using a Hitachi U-2900 spectrophotometer, and fluorescence measurements were performed using a Hitachi F-7000 fluorimeter. Quartz cuvettes with four polished faces and an optical path of 1 cm were used.

The fluorescence spectra were monitored in the range from 290 to 480 nm, with an excitation wavelength (λ_exc_) at 280 nm. The experiments were performed at 293, 299, 304, 309, and 313 K. The stable temperature of the samples was maintained using a thermal bath from AJMicronal, model AJX-708. All the presented experimental values were averages of three independent experiments. All fluorescence measurements were corrected for the inner filter effect.^39^

BSA (Figure 1S) fluorescence quenching by porphyrins at different temperatures was used to determine the thermodynamic characteristics of interaction of TMPyP and TPPS_4_ with the protein. It has been shown that aggregation between the porphyrin and BSA affects the value of the binding constant.^9^ Thus, the concentrations used for both porphyrins and BSA were chosen to minimize aggregation: with TPPS_4_ concentration ranging from 2.0 × 10^–7^ to 1.6 × 10^–6^ M and TMPyP concentration ranging from 2.5 × 10^–7^ to 2.0 × 10^–6^ M. The absence of aggregates was controlled by registration of the optical absorption spectra of samples. In the fluorescence experiments, aliquots of a porphyrin stock solution were added to the 2.0 μM BSA solution.

After the addition of porphyrin to the albumin solution, a 30 min incubation period was provided for the BSA/porphyrin system to reach thermodynamic equilibrium at room temperature. After the incubation period, the solutions containing BSA and each porphyrin were transferred to the spectrofluorometer, which is equipped with an integrated thermal bath, with the temperature set according to the experimental conditions. Fluorescence spectra were recorded until the system reached thermal equilibrium, a process that required 20 to 30 min.

## Results and Discussions

**Figure 2:**
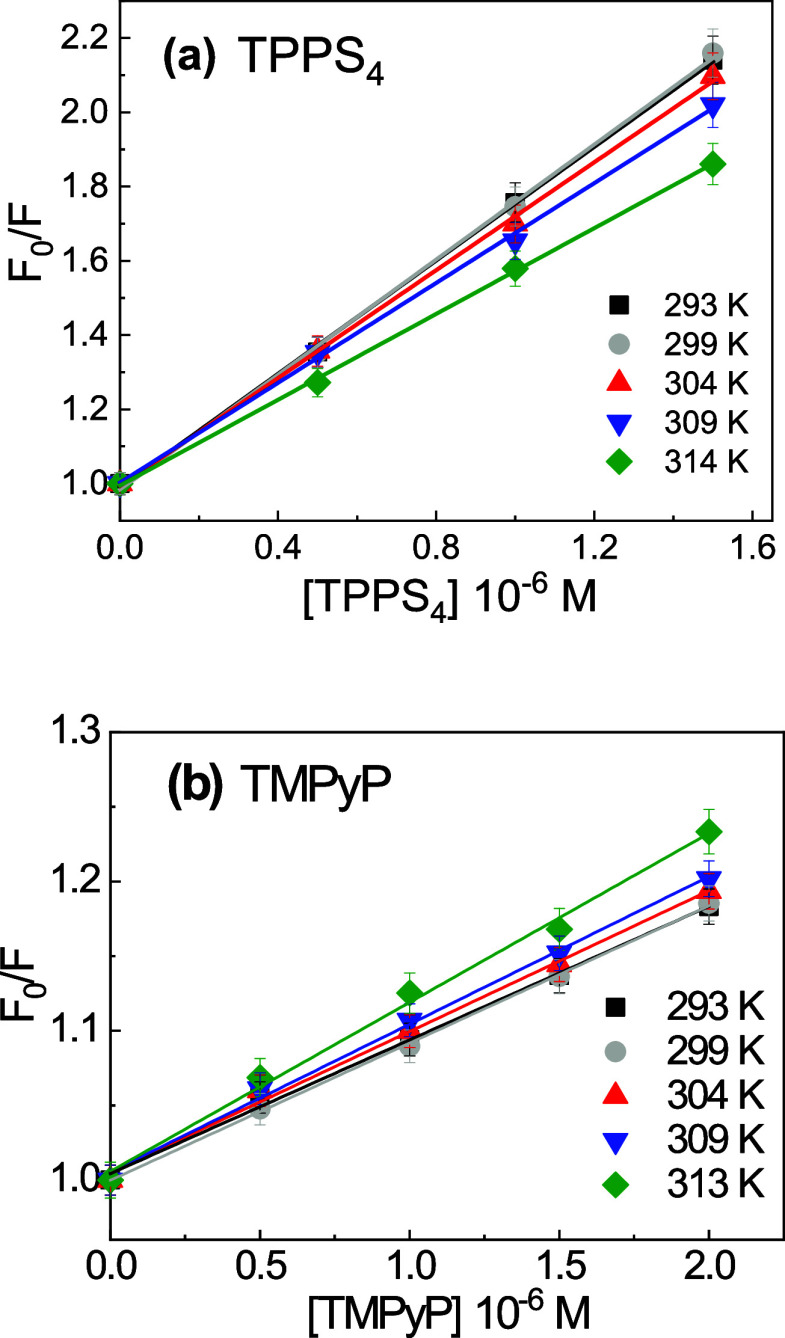
Stern–Volmer plots for BSA fluorescence quenching by TPPS_4_ (a) and TMPyP (b).

The absorption and emission characteristics of albumins are determined by three aromatic amino acid residues: tyrosine (Tyr), tryptophan (Trp), and phenylalanine (Phe).^36^ When albumin is excited at λ_exc_ = 280 nm, the emission observed is mainly due to tryptophan residues, since in addition to its higher absorption at 280 nm, its fluorescence quantum yield (φ_fl_) is also much higher than the φ_fl_ of Tyr and Phe residues. The addition of TPPS_4_ and TMPyP to the BSA solution causes a decrease in its fluorescence intensity (quenching). Figure 2S shows the BSA fluorescence quenching by TPPS_4_ and TMPyP at room temperature.

The quenching of BSA fluorescence by porphyrins was used to analyze the interaction between them. The quenching data were treated using the Stern–Volmer equation:^40^
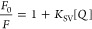
1in which *F* and *F*_0_ are the fluorescence intensities in the presence and absence of the quencher, respectively, and [*Q*] is the quencher concentration. The *K*_SV_ is the Stern–Volmer constant, which reflects the efficiency of the quenching process.

The Stern–Volmer plots for BSA fluorescence quenching by porphyrins at different temperatures are shown in Figure 2.

It was observed that the Stern–Volmer plots at a fixed temperature were linear, and their linearity remained throughout the entire temperature range used. This indicates that each porphyrin quenches a single class of fluorophores in the BSA structure, all of which are equally accessible to the quencher.^40^

Table 1 shows the values of *K*_SV_ for TMPyP and TPPS_4_ at different temperatures. Note that the *K*_SV_ values increase for TMPyP and decrease for TPPS_4_ when the temperature increases.

**Table 1:** Stern–Volmer (*K*_SV_) and Bimolecular (*k*_q_) Quenching Constants of BSA Fluorescence by TPPS_4_ and TMPyP at Different Temperatures

	TPPS_4_		TMPyP	
*T* (K)	*K*_sv_ × 10^5^ M^–1^	*k*_q_ × 10^13^ M^–1^ s^–1^	*K*_SV_ × 10^4^ M^−1^	*k*_q_ × 10^12^ M^–1^ s^–1^
293	7.61 (±0.02)	7.61 (±0.02)	8.96 (±0.06)	1.46 (±0.03)
299	7.70 (±0.01)	7.70 (±0.01)	9.19 (±0.05)	1.51 (±0.02)
303	7.33 (±0.06)	7.33 (±0.06)	9.42 (±0.05)	1.54 (±0.03)
309	6.82 (±0.08)	6.82 (±0.08)	9.89 (±0.08)	1.62 (±0.05)
313	5.76 (±0.06)	5.76 (±0.06)	11.0 (±0.08)	1.80 (±0.05)

Two possible mechanisms of fluorescence quenching exist: *dynamic*, due to collision of a fluorescent molecule with a quencher one, and *static*, due to formation of a nonfluorescent complex between a fluorescent molecule in its ground state and a quencher one.^40^ Sometimes, a parallel implementation of these two mechanisms is considered as a third quenching mechanism. However, when quenching is implemented in parallel by two mechanisms, static and dynamic, the Stern–Volmer dependence is not linear, but quadratic with the concentration of the quencher.^40^ However, in our experiments, we observed linear Stern–Volmer plots for all used temperatures.

The dynamic quenching is controlled by diffusion. Therefore, the maximum value of the bimolecular quenching constant (*k*_q_) is calculated as^40^
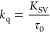
2where τ_0_ is the fluorescence lifetime of the molecule in the absence of the quencher, cannot exceed the diffusion one, whose characteristic value in aqueous solutions is *k*_diff_ ≈ 10^10^ M^–1^ s^–1^.^40^

However, considering τ_0_ for BSA fluorescence equal to 10^–8^ s,^41^ we obtained *k*_q_ by 3 to 4 orders of magnitude greater than *k*_diff_ (Table 1). This indicates that the mechanism of quenching of BSA fluorescence is static for both porphyrins and is associated with the formation of a complex between BSA and porphyrin molecules.^41,42^ In some other studies, the τ_0_ value differs from 10^–8^ s. See, for example, ref (43), where this value is 6.01 × 10^–9^ s. However, the use of this value does not change the above conclusion.

In this case, *K*_SV_ is the binding (complexation) constant *K*_b_. Thus, the system


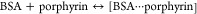


is in thermodynamic equilibrium, and *K*_b_ values can be used to determine thermodynamic parameters.

### Nature of the Forces in the Porphyrin–BSA Interaction

Several types of interaction can be responsible for the molecular complex formation, such as electrostatic interactions, van der Waals forces, hydrophobic effect, and the formation of hydrogen bonds.^44^ In general, determination of thermodynamic parameters is useful to identify the type of interaction responsible for stabilization of a molecular complex. These parameters can be obtained using the linear van’t Hoff equation 3, which is observed if the standard heat capacity *C*^0^ remains unchanged during the process (Δ*C*^0^ = 0):^45^
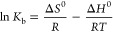
3where Δ*H*^0^ and Δ*S*^0^ are the changes of enthalpy and entropy in the process, respectively, *T* is the absolute temperature, *K*_b_ is the binding constant, and *R* is the gas constant (8.314 J K^–1^ mol^–1^ or 1.987 cal K^–1^ mol^–1^).

Another important thermodynamic parameter is the change in the standard Gibbs free energy (Δ*G*^0^), which indicates whether the process is spontaneous or not. Δ*G*^0^ is defined as
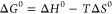
4

The statement about the linearity of the van’t Hoff equation is based on the assumption that standard changes in enthalpy (Δ*H*^0^) and entropy (Δ*S*^0^) do not depend on temperature.

However, in many cases, these parameters are temperature dependent, and the plot of ln *K*_b_ versus 1/*T* is not linear.

If the dependence of ln *K*_b_ on 1/*T* is not linear, it can be approximated by one of two methods, the choice of which depends on whether the change in standard heat capacity Δ*C*^0^ is temperature dependent or independent:^46,47^

(i) if Δ*C*^0^ is invariant with temperature, the dependence of ln *K*_b_ on 1/*T* can be approximated by the logarithmic equation:
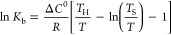
5where *T*_H_ and *T*_S_ are reference temperatures, also called enthalpic (*T*_H_) and entropic (*T*_S_) compensation temperatures, at which Δ*H*^0^ and Δ*S*^0^ are equal to zero.

Expression 5 allows one to estimate the standard enthalpy, entropy, and standard-state Gibbs energy changes from nonlinear van’t Hoff plots by a least-squares fitting procedure as follows:
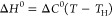
6
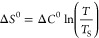
7
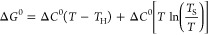
8

(ii) if Δ*C*^0^ is dependent on temperature, the dependence of ln *K*_b_ on 1/*T* can be approximated by the polynomial expression^22,24−26,48^
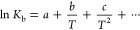
9

The parameters obtained from fitting of the nonlinear van’t Hoff plot by a least-squares procedure can be used to estimate the thermodynamic characteristics according to the equations:
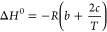
10
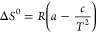
11
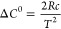
12
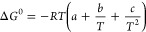
13

**Figure 3:**
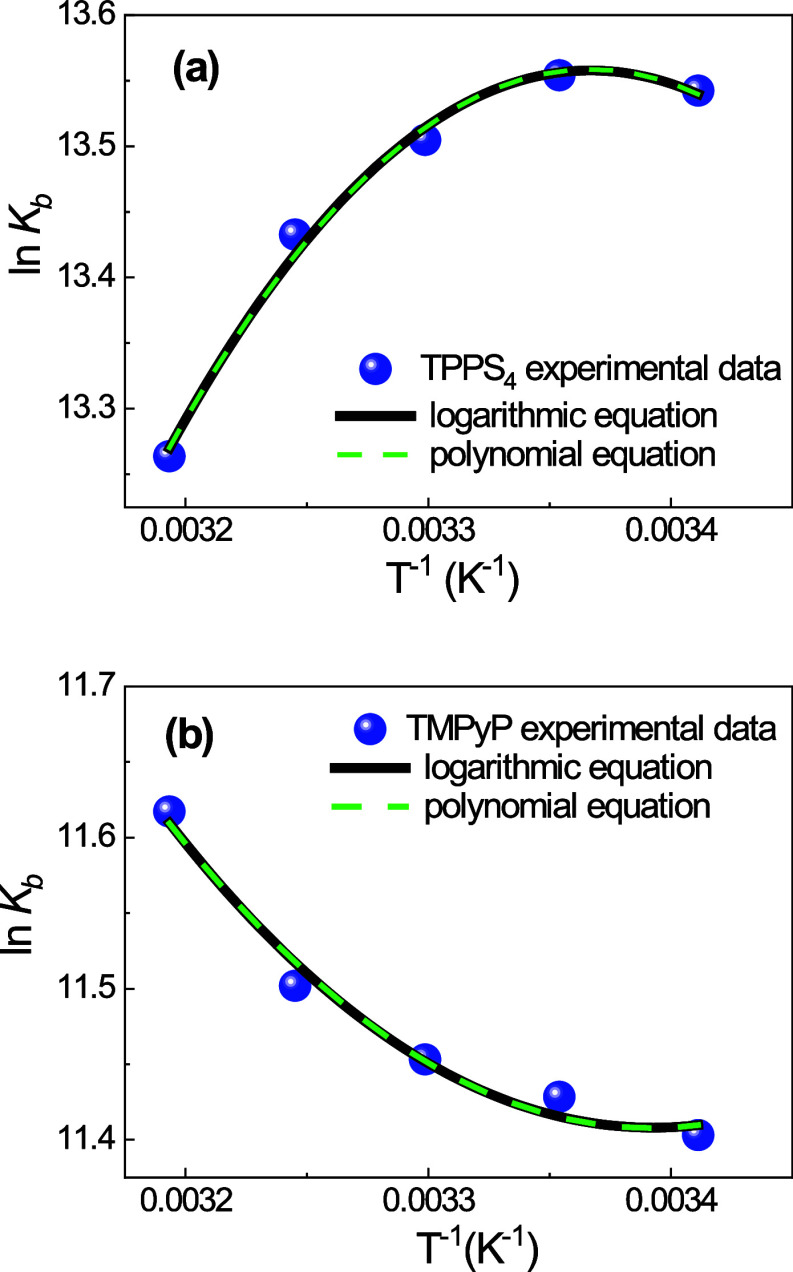
van’t Hoff plots of the logarithm of binding constants ln *K*_b_ with BSA for TPPS_4_ (A) and TMPyP (B) porphyrins. The experimental data and the least-squares fitting using the logarithmic equation 5 (black line, *R*^2^ = 0.98210), which assumes that the standard heat capacity change (Δ*C*^0^) is invariant with temperature and the polynomial equation 9 (green line, *R*^2^ = 0.98109) assuming that the standard heat capacity change is dependent on the temperature.

Figure 3 shows a least-squares fitting of *K*_b_ experimental data using both the logarithmic equation 5 (black line, *R*^2^ = 0.98210) and the polynomial equation 9 (green line, *R*^2^ = 0.98109). As can be seen, both fittings are quite good.

### van’t Hoff Analysis Assuming That the Standard Heat Capacity Change Is Independent of Temperature

The values of *T*_H_ and *T*_S_, obtained from fitting of the data presented in Figure 3, in accordance with the logarithmic equation 5 for standard heat capacity change independent of temperature, Δ*C*^0^ being −50.0 (±6.3) cal mol K^–1^ for TPPS_4_ and 26.8 (±7.5) cal mol K^–1^ for TMPyP are listed in Table 2.

**Table 2:** Enthalpic (*T*_H_) and Entropic (*T*_S_) Compensation Temperatures for the Binding of Porphyrins with BSA in the Case of Standard Heat Capacity (Δ*C*^0^) Dependent and Independent of Temperature

	Δ*C*^0^ independent of temperature		Δ*C*^0^ dependent on temperature	
	TPPS_4_	TMPyP	TPPS_4_	TMPyP
*T*_H_ (K)	297.0 (±0.8)	294.8 (±2.4)	297.1 (±0.3)	294.9 (±0.6)
*T*_S_ (K)	316.9 (±1.8)	266.3 (±9.7)	317.0 (±31)	270.0 (±47)

**Figure 4:**
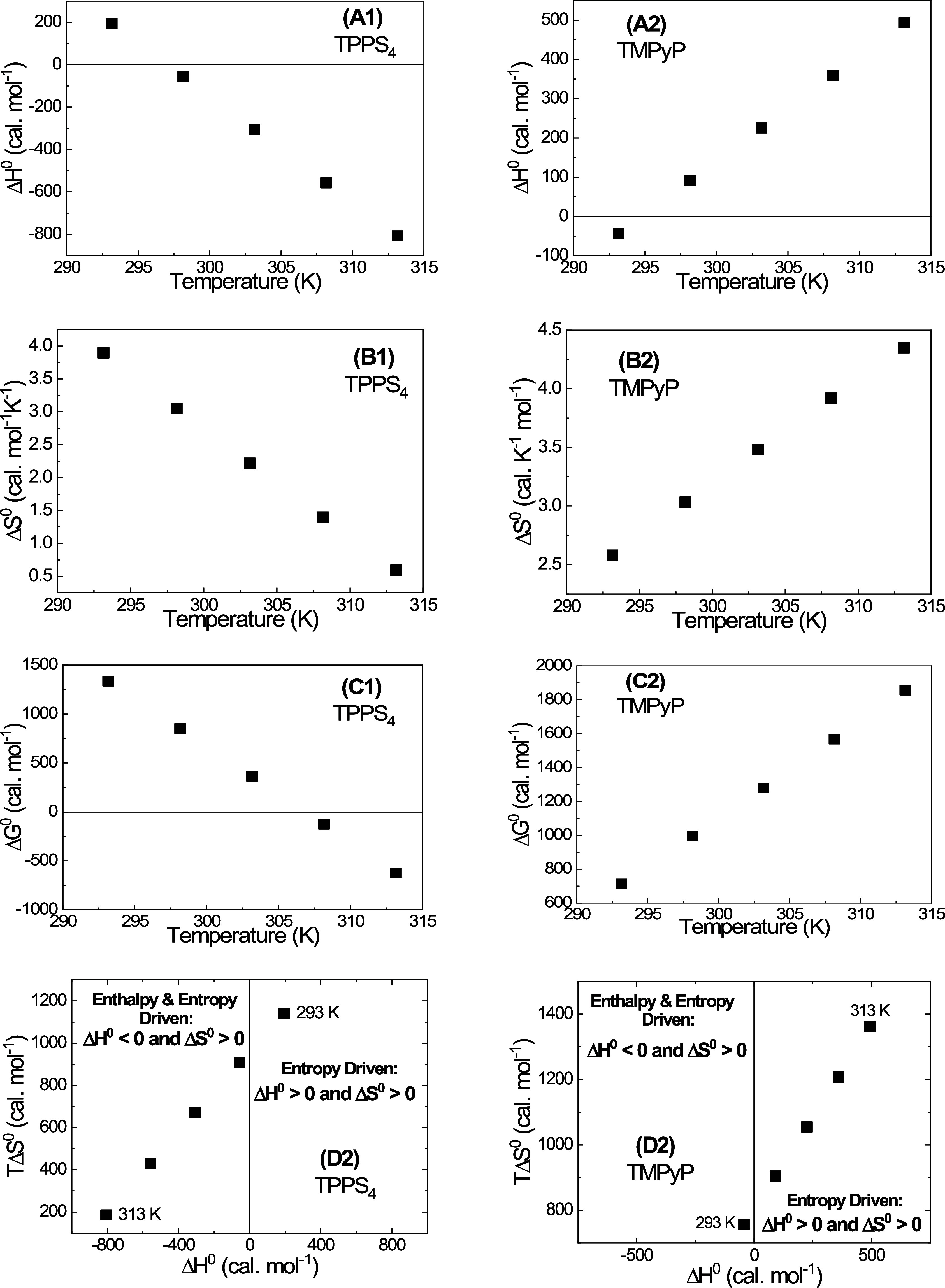
Temperature dependences of thermodynamic parameters of the porphyrin interaction with BSA calculated using eqs 6–8.

The temperature dependences of other thermodynamic parameters of the protein–ligand interaction, determined using these Δ*C*^0^ values, are presented in Figure 4.

It can be seen that there is a fundamental difference between TPPS_4_ and TMPyP. Thus, for TPPS_4_, the standard enthalpy changes (Δ*H*^0^) decrease with the increase in temperature, changing at 295 K from positive to negative with absolute values increasing with temperature (Figure 4A1). This demonstrates an increase of exothermism in the binding process.

The standard entropy changes (Δ*S*^0^) also decrease with the increase in temperature (Figure 4B1), however, being positive over the entire temperature range used. The sign of enthalpy and entropy in the thermodynamic process indicates the type of driving forces involved in the interaction.^22,26^

Thus, for TPPS_4_ for temperatures below 297 K, the binding process is entropically driven (Δ*H*^0^ > 0, Δ*S*^0^ > 0), while above 297 K, it is enthalpy and entropy driven (Δ*H*^0^ < 0, Δ*S*^0^ > 0).

Calculation in accordance with eq 4 demonstrates that Δ*G*^0^ values for TPPS_4_ binding with BSA are positive (Figure 4C1) below 305 K and negative above that temperature.

At the same time, for TMPyP, both Δ*H*^0^ and Δ*S*^0^ increase with increasing temperature (Figure 4A2,B2), Δ*H*^0^ being negative below 297 K and positive above 297 K.

Thus, in contrast with TPPS_4_, the interaction of TMPyP porphyrin with BSA is enthalpy and entropy driven below 297 K (Δ*H*^0^ < 0, Δ*S*^0^ > 0) and entropically driven (Δ*H*^0^ > 0, Δ*S*^0^ > 0) above 297 K.

Calculation in accordance with eq 4 demonstrates that for TMPyP, Δ*G*^*0*^ values are positive (Figure 4C2) over the entire temperature range used.

Thus, according to the assumption that Δ*C*^0^ is independent of the temperature, the process of interaction of TPPS_4_ porphyrin with BSA occurs spontaneously above 305 K and nonspontaneously below 305 K, while for TMPyP porphyrin, this process is nonspontaneous over the entire temperature range used.

### van’t Hoff Analysis Assuming That the Standard Heat Capacity Changes Depend on Temperature

**Figure 5:**
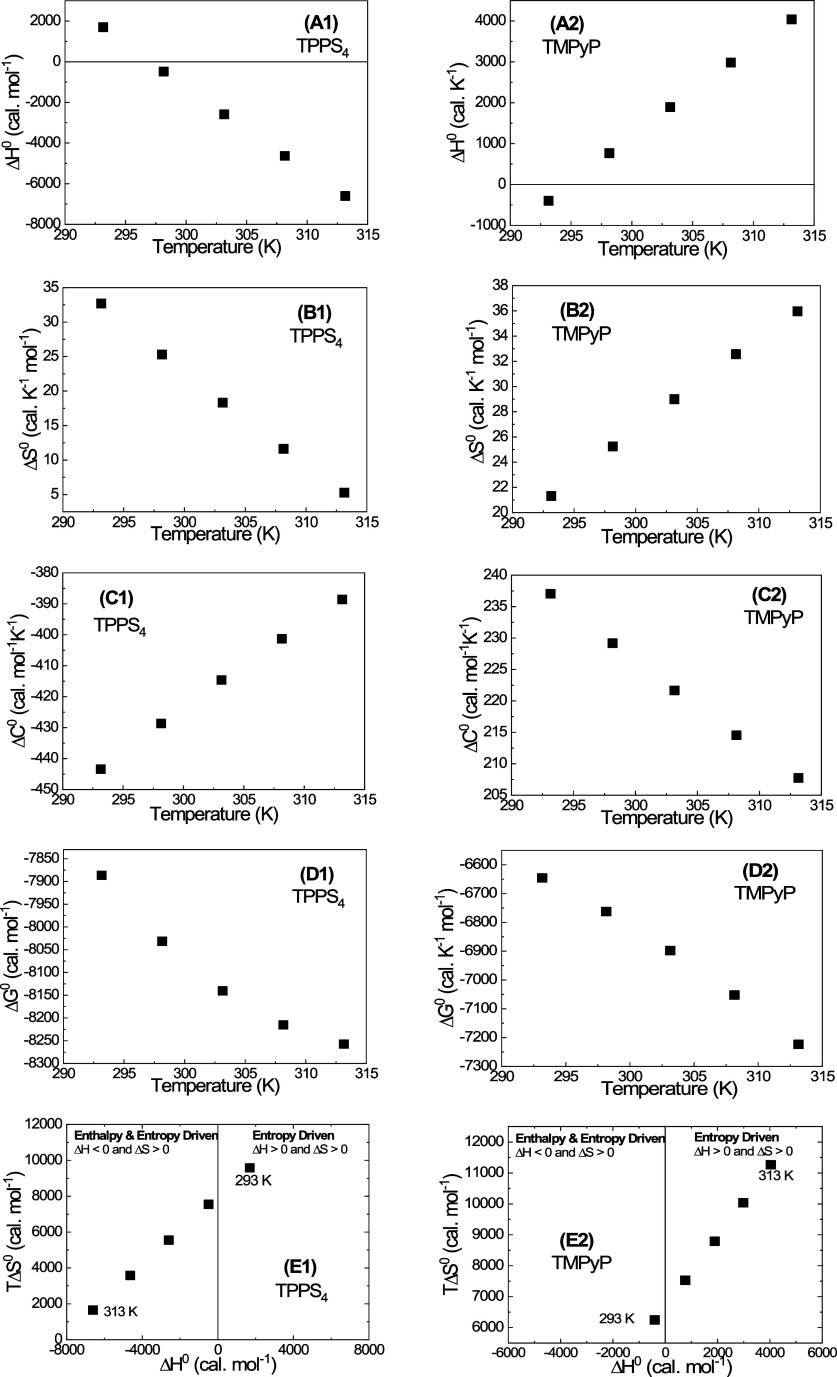
Temperature dependence of the thermodynamic parameters of the porphyrin interaction with BSA calculated using equations evaluated from the least-squares fitting of the parameters *a*, *b*, and *c* calculated using eqs 10–13.

If Δ*C*^0^ depends on temperature, the ln *K*_b_ ↔ *T* experimental dependence should be fitted by a polynomial equation 11.^26,47^Figure 5 illustrates the temperature dependence of thermodynamic characteristics evaluated from the least-squares fitting of the parameters *a*, *b*, and *c* calculated using eq 9 (see Table 1S). As can be seen, the behavior of the standard enthalpy and entropy changes (Figure 5A,B) as well as the driving forces involved in the interaction process are similar to those calculated using the logarithmic equation (Figure 4A,B). The enthalpic (*T*_H_) and entropic (*T*_S_) compensation temperatures are almost equal to those observed for the Δ*C*^0^ temperature-independent model (Table 2).

The analysis of the nature of driving forces involved in the BSA–porphyrin binding within this model shows similarities to those observed in the case of temperature-independent Δ*C*^0^.

In fact, for TPPS_4_ at temperatures below 297 K, the interaction is entropically driven (Δ*H*^0^ > 0, Δ*S*^0^ > 0), while above 297 K, it is enthalpy and entropy driven (Δ*H*^0^ < 0, Δ*S*^0^ > 0) and for TMPyP, it is enthalpy and entropy driven below 297 K (Δ*H*^0^ < 0, Δ*S*^0^ > 0), and entropically driven (Δ*H*^0^ > 0, Δ*S*^0^ > 0) above 297 K.

However, the scales of changes of thermodynamic characteristics in this case are much wider, and Δ*G*^0^ for both TPPS_4_ and TMPyP porphyrins is negative over the entire temperature range (Figure 5), demonstrating the spontaneous nature of the binding process for both porphyrins, which is consistent with high *K*_b_ values. Moreover, a comparison of Δ*G*^0^ for TPPS_4_ and TMPyP shows a higher stability of the TPPS_4_–BSA complex rather than the TMPyP–BSA one, which is in accordance with higher *K*_b_ values for TPPS_4_.

However, the fact that in the case of TPPS_4_, the absolute value of the negative Δ*G*^0^ increases with increasing temperature contradicts the decrease of its *K*_b_ with increasing temperature. Possible reasons for this discrepancy are discussed below.

### Characterization of the Nature of Noncovalent Porphyrin–BSA Interaction

The Δ*C*^0^ signal can be used to characterize the nature of noncovalent interaction in a protein–ligand process. For example, as it has been shown recently, Δ*C*^0^ consists of a negative contribution from exposure of polar groups and a positive one from exposure of nonpolar groups of the amino acid in aqueous solution.^27,28,49^

In the case, when Δ*C*^0^ is independent of the temperature, the signal of Δ*C*^0^ obtained in the data fitting in accordance with the logarithmic equation 5 is negative for TPPS_4_ porphyrin and positive for TMPyP porphyrin.

Thus, based on this model, it can be concluded that the process of noncovalent interaction of the BSA↔TPPS_4_ is likely determined by hydration of polar groups of the protein, while for BSA↔TMPyP interaction, most likely, the interaction is governed by hydration of nonpolar groups of the protein.

In the case of Δ*C*^0^ dependent on temperature, the fitting in accordance with the polynomial equation (9) shows that the Δ*C*^0^ is negative for TPPS_4_↔BSA interaction over the whole temperature range used and increases linearly with increasing temperature, while for the TMPyP↔BSA interaction, Δ*C*^0^ is positive and decreases with increasing temperature (Figure 5C1,C2).

Thus, this result is consistent with the conclusion derived from the first model that the TPPS_4_ interaction with BSA is likely mediated by hydration of the protein polar groups, whereas the BSA↔TMPyP interaction is mediated by hydration of its nonpolar groups.

Furthermore, the fact that for the BSA↔TPPS_4_ interaction Δ*C*^0^ increases linearly with the increasing temperature means that increasing temperature promotes greater hydration of nonpolar groups in the protein. Whereas, in the case of BSA↔TMPyP interaction, Δ*C*^0^ decreases with increasing temperature. This shows that increasing temperature tends to decrease the hydration of protein polar groups.

Analysis based on both models leads to the conclusion that binding sites for TPPS_4_ and TMPyP are different and they are localized at different points in the BSA structure.

### Identification of Possible Localization of Binding Sites for Porphyrins

Despite high albumin versatility in binding different types of molecules, this does not mean they bind randomly to the protein surface, but albumin possesses highly specific binding sites for a great variety of molecules.^50^

The structure of BSA (Figure 1S) shows three large structurally similar domains, called I, II, and III, and each domain contains two subdomains, classified as A and B, respectively. Bovine serum albumin has two tryptophan fragments called Trp-134 and Trp-212, located in the subdomains IA and IIA, respectively.^11,51−54^ Most of the compounds transported by BSA bind at sites within the IB, IIA, and IIIA subdomains, known as the Heme site, the Sudlow I site, and the Sudlow II site, respectively.^55^ The Heme site is a hydrophobic cavity located close to Trp-134 on the protein surface (hydrophilic contact).^56^ On the other hand, the Sudlow I site is a framework where the distribution of amino acid residues leads to a hydrophobic surface on one side and a positively charged surface on the other. The Sudlow I site is characterized as the binding site of large heterocyclic compounds. It is a neighbor of the tryptophan Trp-212, hidden inside (hydrophobic contact).^57^ The Sudlow site II, located in subdomain IIIA away from Trp-212 and Trp-134, has a higher affinity for small aromatic carboxylic acids.^56^ Crystallographic data show that in BSA tryptophan, Trp-212 (Trp 214 for HSA) is spatially located in the vicinity of the Sudlow I site, while tryptophan Trp-134 is close to the Heme site.^52−54^

Three main binding sites in the structure of serum albumin, namely the Heme site, Sudlow site I, and Sudlow site II, show the greatest affinity for porphyrins and appear to be the most likely binding sites.^11,53−57^

An effective method to determine localization of a bound compound in an albumin structure is Förster resonance energy transfer (FRET) analysis of the fluorescence quenching.^58^

FRET occurs between a donor molecule in its electronic excited state and an acceptor molecule in its ground state under the following valid conditions:^59^ (i) the donor has intense fluorescence, and the acceptor has intense optical absorption in UV, visible, or IR; (ii) there is an overlap between the emission spectrum of the donor and the absorption spectrum of the acceptor.

According to a classical model, FRET can be due to the oscillating dipole–dipole interaction between the donor and the acceptor. Typically, the maximum distance between the donor and acceptor at which FRET continues to be effective is about 10 nm.

According to Förster’s theory, the quenching constant of a donor fluorescence by an acceptor (*k*_T_) is equal to^60^
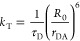
14where *τ*_D_ is the lifetime of the donor excited state in the absence of an acceptor, *r*_DA_ is the distance between the donor and the acceptor, and *R*_0_ is the Förster distance, that is, the donor–acceptor distance at which *k*_T_ = 1/τ_D_. *R*_0_ is determined from the equation:
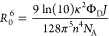
15where κ^2^ is the orientation factor (the mean value of κ^2^ = 2/3 is generally considered for completely random orientation of electric dipoles),^60^*Φ*_D_ is the quantum yield of the donor fluorescence in the absence of the acceptor, *J* = [∫*f*_D_(λ)ε_A_(λ)λ^4^ dλ] is the factor of energy transition, calculated as an integral of the overlap between the emission spectrum of the donor *f*_D_(λ) and the absorption spectrum of the acceptor ε_A_(λ), multiplied by λ^4^, *n* is the refractive index of the medium, and *N*_A_ is the Avogadro number.

Using the *k*_T_ value, obtained experimentally from the donor fluorescence quenching, and *R*_0_, calculated from experimental spectroscopic data, *r*_DA_ can be calculated as
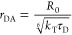
16

The *R*_0_ value was calculated using the emission spectrum of the protein and the absorption spectrum of porphyrin, as shown in Figure S3, measured at room temperature, BSA *Φ*_D_ = 0.13 and *τ*_D_ = 10 ns.^61,62^

As noted above, two tryptophans in the albumin structure are the main sources of its fluorescence. The linear Stern–Volmer dependence observed for both porphyrins at a constant temperature shows that TPPS_4_ and TMPyP quench the fluorescence of the only tryptophan in the BSA structure. Therefore, using Förster analysis of the albumin fluorescence quenching by porphyrins, it is possible to determine its position in the albumin structure in relation to one of the tryptophan residues.

Calculation in accordance with eq 16 shows that the *r*_DA_ distances between the fluorescent point of BSA (tryptophan, donor) and porphyrin (acceptor) molecules are 8.16(±0.19) Å and 11.12(±0.21) Å for TPPS_4_ and TMPyP, respectively.

This suggests that porphyrins bind to one of the tryptophans in BSA. Based on this data, the Sudlow II site (subdomain IIIA) can be excluded as a possible candidate for ligand–protein interaction since, as noted above, crystallographic data show that Trp-212 is close to the Sudlow I site, while Trp-134 is close to the Heme site.^52−54^ In addition, the Sudlow I site selectively binds negatively charged compounds.^57,63^ Therefore, it can be supposed that negatively charged TPPS_4_ may bind to the Sudlow I site (subdomain IIA). On the other hand, TMPyP is positively charged. Therefore, the affinity for binding sites capable of binding negatively charged compounds should be low. Thus, we can exclude the Sudlow I site as a possible binding site for TMPyP. We know that the Heme site has a high affinity for porphyrins and porphyrin-like compounds^55,57^ and considering the distance between tryptophan and TMPyP, 11.12 Å, it can be assumed that TMPyP is bound in the Heme site (between the subdomain IA and IB). These findings are in accordance with those on interaction of porphyrins with serum albumin published elsewhere.^53,64−70^

The assumption that TPPS_4_ and TMPyP porphyrins bind with different binding sites is consistent with the results of thermodynamic analysis, where it was shown that TPPS_4_ and TMPyP bound to BSA are in different chemical environments.

On the other hand, binding to different sites could explain different effects of temperature on the binding constant values for these two porphyrins. It is well-known that changes in environmental temperature cause conformational changes in albumin molecules.^71−74^ Therefore, any change in temperature, on the one hand, can change the accessibility of binding sites, affecting the binding constant; on the other hand, it changes the distance between the fluorophore (tryptophan) and the quencher (porphyrin), thus affecting the value of the tryptophan fluorescence quenching constants. This may explain the contradiction between the increase in Δ*G*^0^ absolute values with increasing temperature for the TPPS_4_–BSA interaction, calculated within the temperature-dependent Δ*C*^0^ model, and the observed decrease in the fluorescence quenching constant of BSA by TPPS_4_ with increasing temperature. One can assume that regardless of the increase in the binding constant of TPPS_4_ to BSA with increasing temperature, an increase in the distance between the bound TPPS_4_ molecule and the tryptophan residue reduces the quenching constant of tryptophan fluorescence of TPPS_4_.

In any case, to confirm the proposed hypotheses, a more detailed study is required, which we plan to perform in the future.

It should be expected that structural changes in the BSA molecule due to temperature changes can cause changes in Δ*C*^0^. This suggests that of the two models used in this work, namely, the assumption that Δ*C*^0^ is independent or dependent on temperature, the second model is preferable. Moreover, the positive Δ*G*^0^ obtained from the interaction of TMPyP with BSA within the framework of the first model is doubtful, since this means that the process of TMPyP binding with BSA is nonspontaneous, which contradicts high values of binding constants (bimolecular constants, this process significantly exceeds the diffusion limit, Table 1).

Based on these statements, we consider the second model, namely Δ*C*^0^, which depends on temperature, more adequate.

## Conclusion

Thermodynamic characteristics of interaction between bovine serum albumin (BSA) and negatively charged *meso*-tetrakis(*p*-sulfonatophenyl) (TPPS_4_) and positively charged *meso*-tetrakis(4-methylpyridyl) (TMPyP) porphyrins were analyzed based on the nonlinear van’t Hoff dependence of logarithm of BSA–porphyrin binding constants (ln(*K*_b_)) as a function of inverse absolute temperature (1/*T*). The constants were determined via BSA fluorescence quenching by porphyrins. The analysis demonstrates that the difference in charge of the side porphyrin groups leads to a difference in localization of TPPS_4_ and TMPyP in the BSA structure. The comparison with the data obtained from the Förster fluorescence quenching (FRET) theory and with literature data led to the conclusion that the negatively charged TPPS_4_ is bound to the Sudlow I site (subdomain IIA), while the positively charged TMPyP is bound in the Heme site (between the subdomains IA and IB). Based on the analysis of the standard heat capacity (Δ*C*^0^) changes, we conclude that binding of TPPS_4_ promotes hydration of nonpolar groups in the protein, which increases with rising temperature, while binding of TMPyP decreases the hydration of polar groups of the protein, the effect increasing with rising temperature. In fact, it should be expected that structural changes in the BSA molecule due to changes in the temperature can cause changes in Δ*C*^0^. Moreover, the BSA structural changes can affect the distance between the porphyrin binding site and the tryptophan fluorophore, thus affecting the FRET distance and producing the difference between the real binding constant and that obtained by BSA fluorescence quenching.

The information obtained in the present work may be useful for elucidating the mechanisms of interaction of porphyrins with albumins and explaining the effects of this interaction on the effectiveness of porphyrins as PS for PDT and FP for FD.

## Supporting Information Available

Representation of bovine serum albumin (BSA) structure, highlighting the location of tryptophan residues; BSA fluorescence quenching with increasing concentrations of studied porphyrins at room temperature; spectral overlaps between BSA fluorescence emission and the molar absorption spectra of porphyrins as well as the fluorescence emission of the studied porphyrins; parameters obtained from fitting of the nonlinear van’t Hoff plot by a least-squares procedure can be used to estimate the thermodynamic characteristics (PDF)

## References

[ref1] Dougherty T. J.; Gomer C. J.; Henderson B. W.; Jori G.; Kessel D.; Korbelik M.; Moan J.; Peng Q. Photodynamic Therapy. JNCI 1998, 90, 889–905. 10.1093/jnci/90.12.889. 9637138 PMC4592754

[ref2] Dolmans D.; Fukumura D.; Jain R. Photodynamic therapy for cancer. Nat. Rev. Cancer 2003, 3, 380–387. 10.1038/nrc1071. 12724736

[ref3] Ribeiro A. P.; Andrade M. C.; Bagnato V. S.; Vergani C. E.; Primo F. L.; Tedesco A. C.; Pavarina A. C. Antimicrobial photodynamic therapy against pathogenic bacterial suspensions and biofilms using chloro-aluminum phthalocyanine encapsulated in nanoemulsions. Lasers Med. Sci. 2015, 30, 549–559. 10.1007/s10103-013-1354-x. 23748800

[ref4] Silva E. F. F.; Schaberle F. A.; Monteiro C. J. P.; Dabrowski J. M.; Arnaut L. G. The challenging combination of intense fluorescence and high singlet oxygen quantum yield in photostable chlorins – a contribution to theranostics. Photochem. Photobiol. Sci. 2013, 12, 1187–1192. 10.1039/c3pp25419d. 23584281

[ref5] Zhao C.; Ur Rehman F.; Yang Y.; et al. Bio-imaging and Photodynamic Therapy with Tetra Sulphonatophenyl Porphyrin (TSPP)-TiO2 Nanowhiskers: New Approaches in Rheumatoid Arthritis Theranostics. Sci. Rep. 2015, 5, 11518 10.1038/srep11518. 26153895 PMC4648397

[ref6] Taratula O.; Schumann C.; Naleway M. A.; Pang A. J.; Chon K. J.; Taratula O. A multifunctional theranostic platform based on phthalocyanine-loaded dendrimer for image-guided drug delivery and photodynamic therapy. Mol. Pharmaceutics 2013, 10, 3946–3958. 10.1021/mp400397t. 24020847

[ref7] Kratz F.; Beyer U. Serum Proteins as Drug Carriers of Anticancer Agents: A Review. Drug Delivery 1998, 5, 281–299. 10.3109/10717549809065759. 19569996

[ref8] Kubat P.; Lang K.; Anzenbacher Jr P. Modulation of porphyrin binding to serum albumin by pH. Biochim. Biophys. Acta 2004, 1670, 40–48. 10.1016/j.bbagen.2003.10.011. 14729140

[ref9] Borissevitch I. E.; Tominaga T. T.; Imasato H.; Tabak M. Fluorescence and optical absorption study of interaction of two water soluble porphyrins with bovine serum albumin. The role of albumin and porphyrin aggregation. J. Lumin. 1996, 69 (2), 65–76. 10.1016/0022-2313(96)00037-3.

[ref10] Borissevitch I. E.; Tominaga T. T.; Schmitt C. C. Photophysical studies on the interaction of two water-soluble porphyrins with bovine serum albumin. Effects upon the porphyrin triplet state characteristics. J. Photochem. Photobiol., A 1998, 114 (3), 201–207. 10.1016/S1010-6030(98)00216-0.

[ref11] Rozinek S. C.; Thomas R. J.; Brancaleon L.Biophysical characterization of the interaction of human albumin with an anionic porphyrin. Biochem. Biophys. Rep. 2016, 7, 295–302. 10.1016/j.bbrep.2016.07.014. 28955918 PMC5613655

[ref12] Codognato D. C. K.; Pena F. S.; dos Reis E. R.; Ramos A. P.; Borissevitch I. E. Effects of serum albumin on the photophysical characteristics of synthetic and endogenous protoporphyrin IX. Braz. J. Med. Biol. Res. 2022, 55, e12272 10.1590/1414-431X2022e12272. 36197413 PMC9529045

[ref13] Gonçalves P. J.; Bezerra F. C.; Almeida L. M.; Alonso L.; Souza G. R. L.; Alonso A.; Zílio S. C.; Borissevitch I. E. Effects of bovine serum albumin (BSA) on the excited-state properties of meso-tetrakis(sulfonatophenyl) porphyrin (TPPS_4_). Eur. Biophys. J. 2019, 48, 721–729. 10.1007/s00249-019-01397-w. 31549191

[ref14] Costa-Tuna A.; Chaves O. A.; Loureiro R. J. S.; Pinto S.; Pina J.; Serpa C. Interaction between a water-soluble anionic porphyrin and human serum albumin unexpectedly stimulates the aggregation of the photosensitizer at the surface of the albumin. Int. J. Bio. Macromol. 2024, 255, 128210 10.1016/j.ijbiomac.2023.128210. 37992936

[ref15] Abboud R.; Charcosset C.; Greige-Gerges H. Interaction of triterpenoids with human serum albumin: A review. Chem. Phys. Lipids 2017, 207, 260–270. 10.1016/j.chemphyslip.2017.05.011. 28576384

[ref16] Siddiqui S.; Ameen F.; Ur Rehman S.; Sarwar T.; Tabish M. Studying the interaction of drug/ligand with serum albumin. J. Mol. Liq. 2021, 336, 116200 10.1016/j.molliq.2021.116200.

[ref17] Liu H.-Y.; Xu Z.-H.; Liu X.-H.; Pin-Xian X.; Zeng Z. Analysis of Binding Interaction between Bovine Serum Albumin and the Cobalt(II) Complex with Salicylaldehyde-2-phenylquinoline-4-carboylhydrazone. Chem. Pharm. Bull. 2009, 57, 1237–1242. 10.1248/cpb.57.1237. 19881274

[ref18] Hou H.-N.; Qi Z.-D.; OuYang Y.-W.; Liao F.-L.; Zhang Y.; Liu Y. Studies on interaction between Vitamin B12 and human serum albumin. J. Pharm. Biomed. Anal. 2008, 47, 134–139. 10.1016/j.jpba.2007.12.029. 18261869

[ref19] Maiti T. K.; Ghosh K. S.; Samanta A.; Dasgupta S. The interaction of silibinin with human serum albumin: A spectroscopic investigation. J. Photochem. Photobiol., A 2008, 194, 297–307. 10.1016/j.jphotochem.2007.08.028.

[ref20] Shahabadi N.; Khodaei M. M.; Kashanian S.; et al. Study on the interaction of a copper(II) complex containing the artificial sweetener aspartame with human serum albumin. Mol. Biol. Rep. 2014, 41, 3271–3278. 10.1007/s11033-014-3189-3. 24481880

[ref21] Osborne J. C. Jr.; Palumbo G.; Brewer H. B. Jr.; Edelhoch H. The Thermodynamics of the Self-Association of the Reduced and Carboxymethylated Form of ApoA-II from the Human High Density Lipoprotein Complex. Biochemistry 1976, 15, 317–320. 10.1021/bi00647a012. 2283

[ref22] Reichenwallner J.; Schwieger C.; Hinderberger D. Probing the Nanoscopic Thermodynamic Fingerprint of Paramagnetic Ligands Interacting with Amphiphilic Macromolecules. Polymers 2017, 9, 324–342. 10.3390/polym9080324. 30971002 PMC6418530

[ref23] Formisano S.; Johnson M. L.; Edelhoch H. Thermodynamics of the self-association of glucagon. Proc. Natl. Acad. Sci. U. S. A. 1977, 74, 3340–3344. 10.1073/pnas.74.8.3340. 269394 PMC431554

[ref24] Galaon T.; David V. Deviation from van’t Hoff dependence in RP-LC induced by tautomeric interconversion observed for four compounds. J. Sep. Sci. 2011, 34, 1423–1428. 10.1002/jssc.201100029. 21538875

[ref25] Tanase M.; Soare A.; David V.; Moldoveanu S. C. Sources of Nonlinear van’t Hoff Temperature Dependence in High-Performance Liquid Chromatography. ACS Omega 2019, 4, 19808–19817. 10.1021/acsomega.9b02689. 31788613 PMC6882149

[ref26] Vieira E. D.; Basso L. G. M.; Costa-Filho A. J. Non-linear van’t Hoff behavior in pulmonary surfactant model membranes. Biochim. Biophys. Acta, Biomembr. 2017, 1859, 1133–1143. 10.1016/j.bbamem.2017.03.011. 28336314

[ref27] Privalov P. L.; Makhatadze G. I. Contribution of Hydration and Non-covalent Interactions to the Heat Capacity Effect on Protein Unfolding. J. Mol. Biol. 1992, 224, 715–723. 10.1016/0022-2836(92)90555-X. 1314903

[ref28] Milardi D.; Fasone S.; La Rosa C.; Grasso D. Contributions of polar and apolar groups to the thermodynamic stability of azurin. Il Nuovo Cimento D 1996, 18, 1347–1354. 10.1007/BF02453268.

[ref29] Garcia-Sampedro A.; Tabero A.; Mahamed I.; Acedo P. Multimodal use of the porphyrin TMPyP: From cancer therapy to antimicrobial applications. J. Porphyrins Phthalocyanines 2019, 23, 11–27. 10.1142/S1088424619500111.

[ref30] Juarranz A.; Villanueva A.; Díaz V.; Cañete M. Photodynamic effects of the cationic porphyrin, mesotetra(4N-methylpyridyl)porphine, on microtubules of HeLa cells. J. Photochem. Photobiol., B 1995, 27, 47–53. 10.1016/1011-1344(94)07055-S. 7699523

[ref31] Grebenová D.; Cajthamlová H.; Holada K.; Marinov J.; Jirsa M.; Hrkal Z. Photodynamic effects of meso-tetra(4-sulfonatophenyl) porphine on human leukemia cells HEL and HL60, human lymphocytes and bone marrow progenitor cells. J. Photochem. Photobiol., B 1997, 39, 269–278. 10.1016/S1011-1344(97)00017-1. 9253201

[ref32] Almeida L. M.; Zanoelo F. F.; Castro K. P.; Borissevitch I. E.; Soares C. M. A.; Gonçalves P. J. Cell survival and altered gene expression following photodynamic inactivation of Paracoccidioides brasiliensis. Photochem. Photobiol. 2012, 88 (4), 992–1000. 10.1111/j.1751-1097.2012.01112.x. 22332981

[ref33] Teles A. V.; Oliveira T. M. A.; Bezerra F. C.; Alonso L.; Alonso A.; Borissevitch I. E.; Gonçalves P. J.; Souza G. R. L. Photodynamic inactivation of Bovine herpesvirus type 1 (BoHV-1) by porphyrins. J. Gen. Virol. 2018, 99, 1301–1306. 10.1099/jgv.0.001121. 30058992

[ref34] Borissevitch I. E.; Silveira-Alves J. E.; Almeida C. G. L.; Souza G. R. L.; Sokolov S. S.; Gonçalves P. J. An Alternative Method to Determine the Quantum Yield of the Excited Triplet State Using Laser Flash Photolysis. Photonics 2023, 10, 409 10.3390/photonics10040409.

[ref35] Gonçalves P. J.; De Boni L.; Neto N. M. B.; Rodrigues J. J.; Zílio S. C.; Borissevitch I. E. Effect of protonation on the photophysical properties of meso-tetra(sulfonatophenyl) porphyrin. Chem. Phys. Lett. 2005, 407, 236–241. 10.1016/j.cplett.2005.03.100.

[ref36] Gonçalves P. J.; Borissevitch I. E.; Zílio S. C. Effect of protonation on the singlet–singlet excited-state absorption of meso-tetrakis(p-sulphonatophenyl) porphyrin. Chem. Phys. Lett. 2009, 469, 270–273. 10.1016/j.cplett.2008.12.067.

[ref37] Gonçalves P. J.; Franzen P. L.; Correa D. S.; Almeida L. M.; Takara M.; Ito A. S.; Zílio S. C.; Borissevitch I. E. Effects of environment on the photophysical characteristics of mesotetrakis methylpyridiniumyl porphyrin (TMPyP). Spectrochim. Acta, Part A 2011, 79 (5), 1532–1539. 10.1016/j.saa.2011.05.012. 21641855

[ref38] Pace C. N.; Vajdos F.; Fee L.; Grimsley G.; Gray T. How to measure and predict the molar absorption coefficient of a protein. Protein Sci. 1995, 4, 2411–2423. 10.1002/pro.5560041120. 8563639 PMC2143013

[ref39] Borissevitch I. E. More about the inner filter effect: Corrections of Stern–Volmer fluorescence quenching constants are necessary at very low optical absorption of the quencher. J. Lumin. 1999, 81 (3), 219–224. 10.1016/S0022-2313(98)00063-5.

[ref40] Lakowicz J. R. Principles of Fluorescence Spectroscopy, 3rd ed.; Springer Science+Business Media, LLC, 2006; pp 878–881.

[ref41] Ni Y.; Liu G.; Kokot S. Fluorescence spectrometric study on the interactions of Isoprocarb and sodium 2-isopropylphenate with bovine serum albumin. Talanta 2008, 76, 513–521. 10.1016/j.talanta.2008.03.037. 18585315

[ref42] Zhao L.; Liu R.; Zhao X.; Yang B.; Gao C.; Hao X.; Wu Y. New strategy for the evaluation of CdTe quantum dot toxicity targeted to bovine serum albumin. Sci. Total Environ. 2009, 407, 5019–5023. 10.1016/j.scitotenv.2009.05.052. 19540569

[ref43] Chaves O. A.; Loureiro R. J. S.; Serpa C.; Cruz P. F.; Ferreira A. B. B.; Netto-Ferreira J. C. Increasing the polarity of β-lapachone does not affect its binding capacity with bovine plasma protein. Int. J. Biol. Macromol. 2024, 263 (2), 130279 10.1016/j.ijbiomac.2024.130279. 38401585

[ref44] Karshikoff A. Non-Covalent Interactions in Proteins World Scientific Publishing Co. Pte. Ltd. 2006 1–176 10.1142/9781860948817_0001

[ref45] Atkins P. ATKINS’ Physical Chemistry, 8th ed.; Oxford University Press, 2006; pp. 200–239.

[ref46] Baldwin R. L. Temperature dependence of the hydrophobic interaction in protein folding. Proc. Natl. Acad. Sci. U. S. A. 1986, 83 (21), 8069–8072. 10.1073/pnas.83.21.8069. 3464944 PMC386868

[ref47] Haidacher D.; Vailaya A.; Horváth C. Temperature effects in hydrophobic interaction chromatography. Proc. Natl. Acad. Sci. U. S. A. 1996, 93, 2290–2295. 10.1073/pnas.93.6.2290. 8637865 PMC39788

[ref48] Yamaguchi A.; Ishii A.; Kamijo T. Influence of ionic strength and temperature on adsorption of tetrakis-N-methylpyridyl porphyrin onto mesoporous silica. Colloids Surf. 2022, 655, 130262 10.1016/j.colsurfa.2022.130262.

[ref49] Murphy K. P.; Gill S. J. Solid Model Compounds and the Thermodynamics Protein Unfolding. J. Mol. Biol. 1991, 222, 699–709. 10.1016/0022-2836(91)90506-2. 1660931

[ref50] Carter D. C.; Ho J. X. Structure of Serum Albumin. Adv. Protein Chem. 1994, 45, 153–203. 10.1016/S0065-3233(08)60640-3. 8154369

[ref51] Bourassa P.; Hasni I.; Tajmir-Riahi H. A. Folic acid complexes with human and bovine serum albumins. Food Chem. 2011, 129, 1148–1155. 10.1016/j.foodchem.2011.05.094. 25212350

[ref52] Moreira M. B.; Franciscato D. S.; Toledo K. C. F.; de Souza J. R. B.; Nakatani H. S.; de Souza V. R. Invetigation of the Fluorescence Quenching of Bovine and Human Serum Albumin by Ruthenium Complex. Quim. Nova 2015, 38, 227–232. 10.5935/0100-4042.20140315.

[ref53] Lebedeva N. S.; Malkova E. A.; Popova T. E.; Kutyrev A. E.; Syrbu S. A.; Parfenyuk E. V.; Vyugin A. I. Interaction peculiarities of 5,10,15,20-tetrakis(4-N-methylpyridil) tetra iodide porphyrin with albumin. Spectrochim. Acta, Part A 2014, 118, 395–398. 10.1016/j.saa.2013.06.101. 24076455

[ref54] Tian J.; Liu X.; Zhao Y.; Zhao S. Studies on the interaction between tetraphenylporphyrin compounds and bovine serum albumin. Lumin 2007, 22, 446–454. 10.1002/bio.983. 17610308

[ref55] Curry S. Human Serum Albumin - New Insights on its Structural Dynamics, Functional Impacts and Pharmaceutical Applications; Otagiri Masaki, Ed.; Sojo University Publishing Center: Kumamoto, Japan, 2011; pp 1–29.

[ref56] Fanali G.; di Masi A.; Trezza V.; Marino M.; Fasano M.; Ascenzi P. Human serum albumin: From bench to bedside. Mol. Aspects Med. 2012, 33, 209–290. 10.1016/j.mam.2011.12.002. 22230555

[ref57] Komatsu T.; Ohmichi N.; Nakagawa A.; Zunszain P. A.; Curry S.; Tsuchida E. O_2_ and CO Binding Properties of Artificial Hemoproteins Formed by Complexing Iron Protoporphyrin IX with Human Serum Albumin Mutants. J. Am. Chem. Soc. 2005, 127, 15933–15942. 10.1021/ja054819u. 16277537

[ref58] van der Meer B. W. Förster Theory FRET – Förster Resonance Energy Transfer, From Theory to Applications, Medintz Igor; Hildebrandt Niko, Eds.; Wiley-VCH Verlag GmbH & Co. KGaA, 2014, pp 23–59.

[ref59] Hildebrandt N. How to Apply FRET: From Experimental Design to Data Analysis FRET – Förster Resonance Energy Transfer, From Theory to Applications, Medintz Igor; Hildebrandt Niko, Eds.; Wiley-VCH Verlag GmbH & Co. KGaA, 2014, pp 105–156.

[ref60] Hoi H.; Ding Y.; Campbell R. E. FRET with Recently Developed Materials. FRET – Förster Resonance Energy Transfer, From Theory to Applications, Medintz Igor; Hildebrandt Niko, Eds.; Wiley-VCH Verlag GmbH & Co. KGaA, 2014, pp 431–463.

[ref61] Zhao L.; Liu R.; Zhao X.; Yang B.; Gao C.; Hao X.; Wu Y. New strategy for the evaluation of CdTe quantum dot toxicity targeted to bovine serum albumin, Sci. Total. Environ. 2009, 407, 5019–5023. 10.1016/j.scitotenv.2009.05.052. 19540569

[ref62] Yuping Z.; Yongju W.; Na L.; Shenjun Q. Fluorescence quantum yield of human and bovine serum albumin. Chin. J. Anal. Chem. 2004, 32, 779–782.

[ref63] Ghuman J.; Zunszain P. A.; Petitpas I.; Bhattacharya A. A.; Otagiri M.; Curry S. Structural Basis of the Drug-binding Specificity of Human Serum Albumin. J. Mol. Biol. 2005, 353, 38–52. 10.1016/j.jmb.2005.07.075. 16169013

[ref64] Shen G.-F.; Liu T.-T.; Wang Q.; Jiang M.; Shi J.-H. Spectroscopic and molecular docking studies of binding interaction of gefitinib, lapatinib and sunitinib with bovine serum albumin (BSA). J. Photochem. Photobiol., B 2015, 153, 380–390. 10.1016/j.jphotobiol.2015.10.023. 26555641

[ref65] Xiao C.-Q.; Jiang F.-L.; Zhou B.; Lia R.; Liua Y. Interaction between a cationic porphyrin and bovine serum albumin studied by surface plasmon resonance, fluorescence spectroscopy and cyclic voltammetry. Photochem. Photobiol. Sci. 2011, 10, 1110–1117. 10.1039/c1pp05008g. 21431181

[ref66] Lebedeva N. S.; Gubarev Y. A.; Yurina E. S.; Syrbu S. A. Features of interaction of tetraiodide meso-tetra(N-methyl-3-pyridyl) porphyrin with bovine serum albumin. J. Mol. Liq. 2018, 265, 664–667. 10.1016/j.molliq.2018.06.030.

[ref67] Chaves O. A.; Acunha T. V.; Iglesias B. A.; Jesus C. S. H.; Serpa C. Effect of peripheral platinum(II) bipyridyl complexes on the interaction of tetra-cationic porphyrins with human serum albumin. J. Mol. Liq. 2020, 301, 112466 10.1016/j.molliq.2020.112466.

[ref68] Yurina E. S.; Gubarev Y. A.; Lebedeva N. S. A study of protein aggregation activators in molecular complexes of cationic porphyrins and chlorin with BSA. J. Mol. Liq. 2021, 338, 116632 10.1016/j.molliq.2021.116632.

[ref69] Natalya L.; Elena M.; Sergey S.; Yury G.; Dmitry N. Investigation of Interactions Between Cationic and Anionic Porphyrins and BSA in Aqueous Media. Int. J. Biochem. Biophys. 2013, 2 (1), 13–18. 10.13189/IJBB.2014.020103.

[ref70] Waelbroeck M.; Van Obberghen E.; De Meyts P. Thermodynamics of the Interaction of Insulin with Its Receptor. J. Biol. Chem. 1979, 254, 7736–7740. 10.1016/S0021-9258(18)36008-3. 468783

[ref71] Weber P.; Bełdowski P.; Domino K.; Ledziński D.; Gadomski A. Changes of Conformation in Albumin with Temperature by Molecular Dynamics Simulations. Entropy 2020, 22, 405 10.3390/e22040405. 33286179 PMC7516880

[ref72] Wetzel R.; Becker M.; Behlke J.; Billwitz H.; Böhm S.; Ebert B.; Hamann H.; Krumbiegel J.; Lassmann G. Temperature behaviour of human serum albumin. Eur. J. Biochem. 1980, 104 (2), 469–478. 10.1111/j.1432-1033.1980.tb04449.x. 6244951

[ref73] Hao M.; Ji Y.; Wang Y.; Chen Y. AFM Study of Temperature and pH Effects on BSA Structure and Adhesion. In 2019 IEEE International Conference on Manipulation, Manufacturing and Measurement on the Nanoscale (3M-NANO), IEEE, 2019; pp 333–336.

[ref74] Foster J. F. Some aspects of the structure and conformational properties of serum albumin. In Albumin: Structure, Function and Uses, Pergamon, V. M., Rosenoer; Oratz M.; Rothschild M. A., Eds.; Elsevier, 1977; pp 53–84. DOI: 10.1016/B978-0-08-019603-9.50010-7.

