# Supplementary material for: Nonlinear van’t Hoff Behavior in the Interaction of Two Water-Soluble Porphyrins with Bovine Serum Albumin (BSA)
Source: ACS Omega. 2024 Nov 20;9(48):47699–709. doi: 10.1021/acsomega.4c07367 (PMC11618399; doi:10.1021/acsomega.4c07367)
Supplement: Supplementary file 1 — ao4c07367_si_001.pdf [file ao4c07367_si_001.pdf]

Supplementary Material

Non-linear van't Hoff behaviour in interaction of two water-soluble porphyrins with Bovine Serum Albumin (BSA)

Fabio C. Bezerra[1], Ernanni D. Vieira[1],

Pablo J. Gonçalves[1,2,3*] Iouri E. Borissevitch[1,4,**]

[1]*Instituto de Física, Universidade Federal de Goiás, Goiânia, GO, 74690-900, Brazil.*

[2]*Programa de Pós-Graduação em Química, Instituto de Química, Universidade Federal de Goiás, Goiânia, GO, 74690-900, Brazil,*

[3]*Centro de Excelência em Hidrogênio e Tecnologias Energéticas Sustentáveis (CEHTES), Goiânia, GO, 74690-900, Brazil*

[4]*Departamento de Física, Faculdade de Filosofia, Ciências e Letras de Ribeirão Preto, Universidade de São Paulo, Ribeirão Preto - SP, 14040-900, Brazil.*

Authors to whom correspondence should be addressed:

*pablo@ufg.br, and **iourib@ffclrp.usp.br

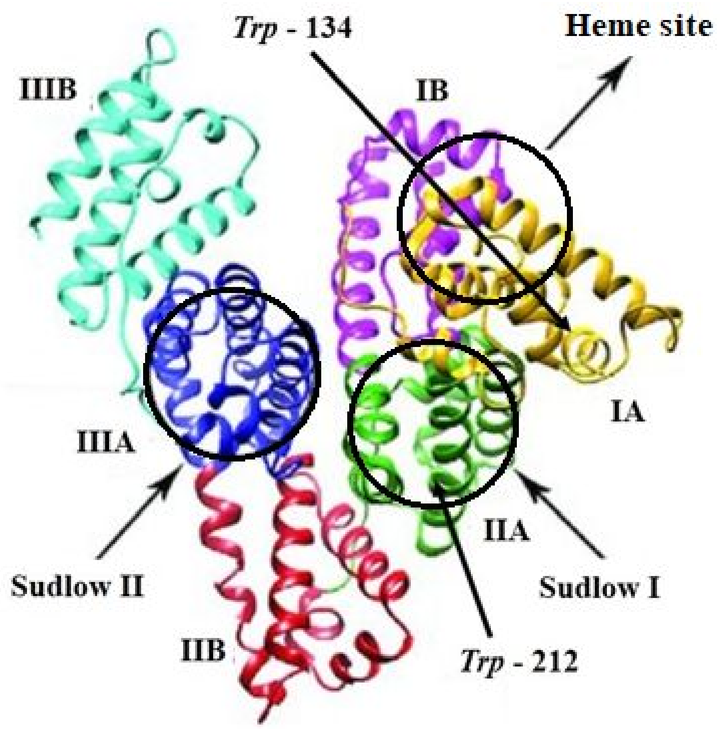

Figure 1S: Representation of bovine serum albumin (BSA), highlighting the location of tryptophan residues. Adapted from [50].

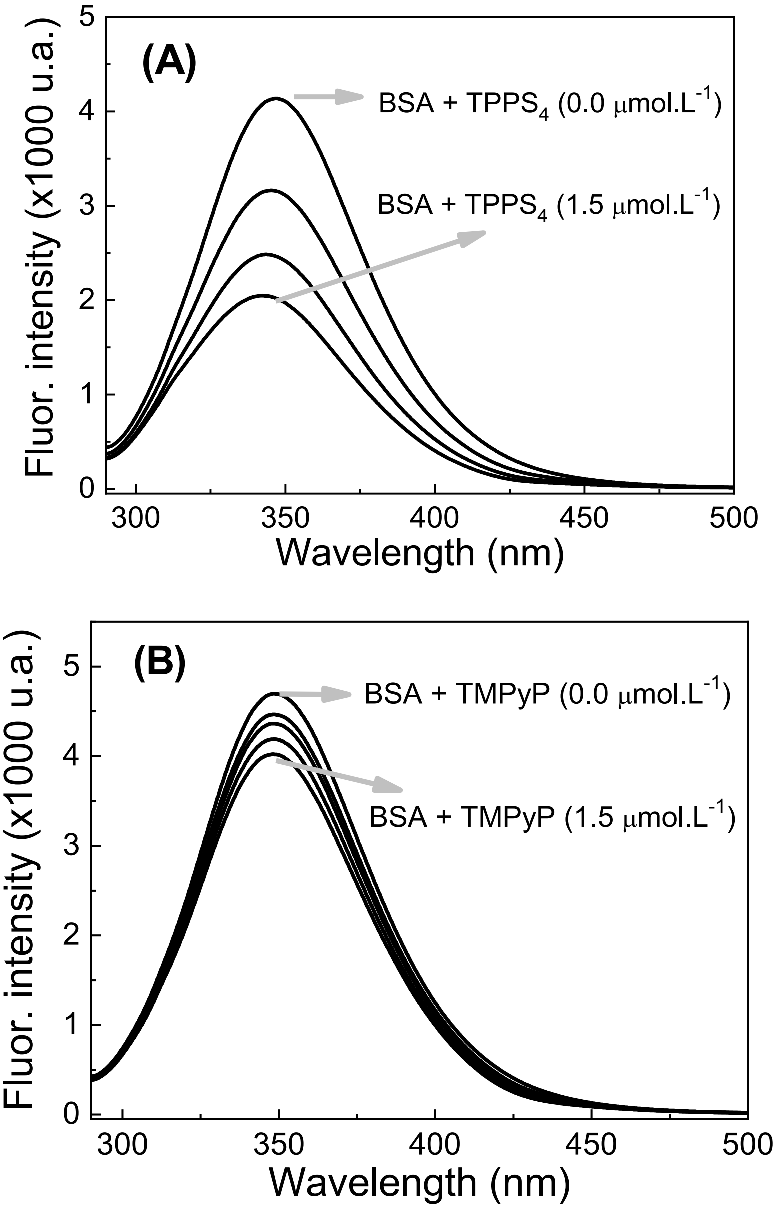

Figure 2S: BSA (2.0 μmol.L^{-1}) fluorescence quenching (λ_{exc}=280 nm) with increasing concentrations of porphyrins $TPPS_4$ (**A**) and TMPyP (**B**) (0.0; 0.5; 1.0 and 1.5 μmol.L^{-1}) at room temperature (299 K).

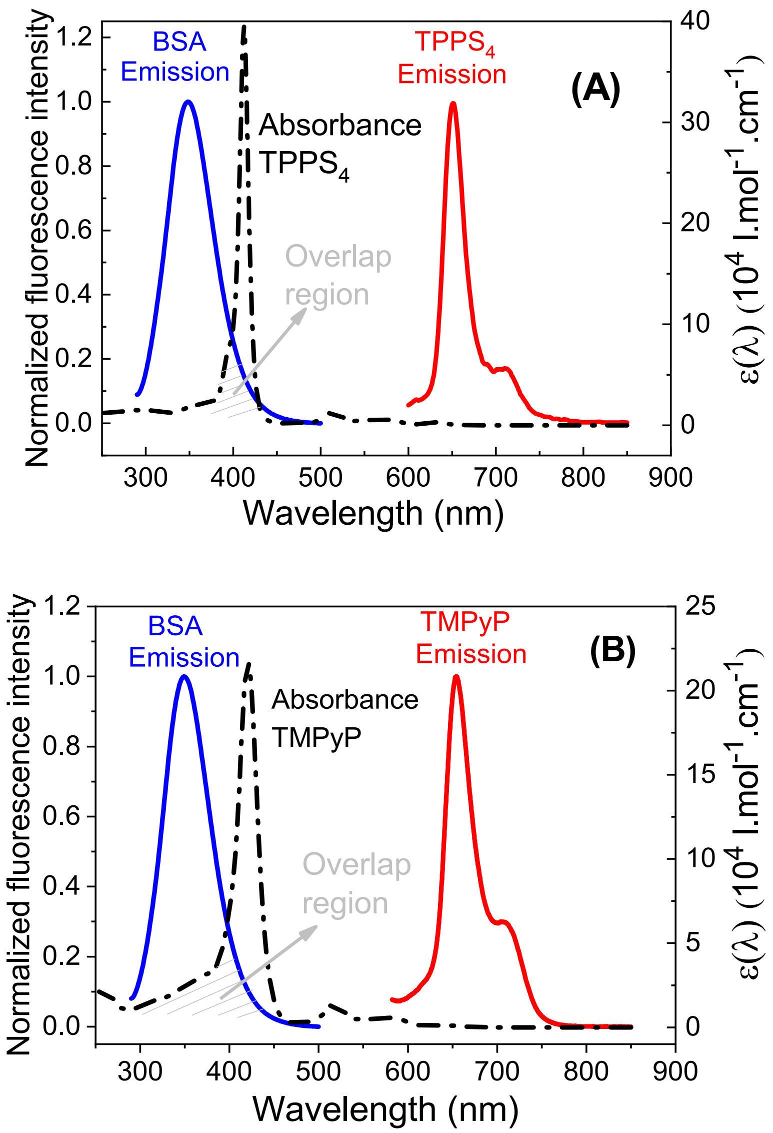

Figure 3S: Spectral overlaps between BSA fluorescence emission (λ_{exc} = 280 nm) and the molar absorption spectra of porphyrins $TPPS_4$ (A) and TMPyP (B), as well as the fluorescence emission of the studied porphyrins (λ_{exc} = 532 nm).

Table 1S: The least-squares fitting of the parameters *a*, *b* and *c* calculated using the equation (11), as described in the text.

Sample	a × 10	(b/K) × 10^3	(b/K^2) × 10^6
$TPPS_4$	7.0(1.6)	-34.8(9.8)	5.1(1.5)
TMPyP	-9.5(1.4)	64.6(8.4)	-9.6(1.3)
